# Pharmacology and toxicology of veterinary isoxazolines: a review

**DOI:** 10.1016/j.ijpddr.2026.100645

**Published:** 2026-04-16

**Authors:** Paulina Markowska-Buńka, Bartosz Rasiński, Hubert Ziółkowski

**Affiliations:** a Department of Pharmacology and Toxicology, Faculty of Veterinary Medicine, University of Warmia and Mazury in Olsztyn, Oczapowskiego 13, 10-718, Olsztyn, Poland; b Waters Sp. z o.o., Wybrzeże Gdyńskie 6B, 01-531, Warszawa, Poland

**Keywords:** Isoxazolines, Pharmacokinetics, Toxicology, Adverse effects, Environmental risk

## Abstract

Isoxazoline derivatives have rapidly become cornerstone ectoparasiticides in veterinary medicine, providing high efficacy and long-lasting protection against fleas, ticks, and other arthropods in companion animals and, more recently, in food-producing species. This review summarizes current knowledge on the chemistry, pharmacology, and toxicology of the main veterinary isoxazolines - fluralaner, afoxolaner, sarolaner, and lotilaner - with particular emphasis on safety profiles and broader One Health considerations. Chemically, these compounds are highly lipophilic, fluorinated molecules with distinctive stereochemistry; the selective use of biologically active S-enantiomers has improved potency while limiting off-target effects. Isoxazolines act through non-competitive antagonism of γ-aminobutyric acid (GABA) - and L-glutamate-gated chloride channels in arthropods, resulting in sustained neuronal hyperexcitation and parasite death, with markedly higher affinity for arthropod than mammalian receptors. Pharmacokinetically, the compounds exhibit variable oral bioavailability but share extensive plasma protein binding, large volumes of distribution, low clearance, and long elimination half-lives, which underlie their prolonged clinical efficacy but also raise concerns regarding tissue accumulation and delayed adverse effects. Controlled studies generally demonstrate wide safety margins; however, post-marketing pharmacovigilance has identified rare but sometimes serious neurological adverse events, particularly in predisposed animals or in the context of impaired efflux transport, such as ABCB1/MDR1 mutations or P-glycoprotein inhibition. The authorization of fluralaner for use in laying hens and evidence of residues in eggs approaching established maximum residue limits emphasize the importance of continued residue monitoring and refinement of withdrawal periods. Moreover, the environmental persistence of isoxazolines and their pronounced toxicity to non-target invertebrates highlight emerging ecotoxicological concerns. Collectively, veterinary isoxazolines represent a highly effective yet complex class of antiparasitic agents whose safe and sustainable use requires integrated pharmacological, toxicological, residue, and environmental risk assessment.

## Highlights

• Isoxazolines provide rapid, long-acting control of fleas, ticks and mites.
• Fluorinated, chiral “-laners” show distinct PK and toxicity profiles.
• They inhibit arthropod GABA- and glutamate-gated chloride channels.
• Post-marketing data report rare neurological AEs despite wide safety margins.
• Residues and environmental persistence raise One Health and resistance concerns.

## Graphical abstract

**Figure:**
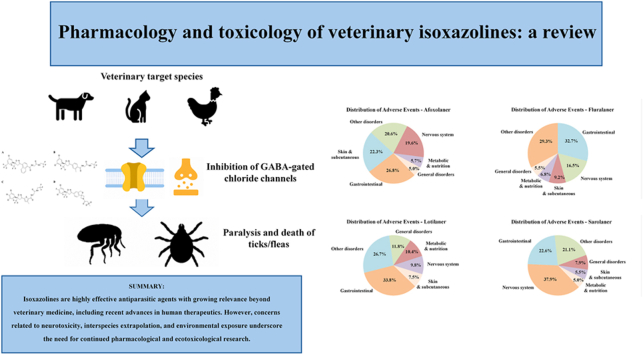

## 1 Introduction

Ectoparasite infestations represent a significant and persistent health challenge in both companion and food-producing animals. They induce direct clinical manifestations, impair animal welfare, and reduce productivity in intensive production systems, leading to substantial economic losses and, in severe cases, mortality (Cosoroabă, 2001; Sárkány et al., 2025). Moreover, they act as important vectors of infectious diseases, affecting humans, companion animals, livestock, and wildlife (Otranto et al., 2013; Pérez de León et al., 2020; Maggi et al., 2022). As only a few vaccines against ectoparasites are currently available, antiparasitic drugs will remain the only effective means of therapy and prevention for many years to come (Selzer and Epe, 2021). These agents are among the most frequently used in veterinary medicine due to the health risks associated with external parasite infestations (Zhou et al., 2022). In recent decades, considerable progress has been made in the development of new ectoparasiticides for companion animals, particulary dogs and cats, both in terms of active ingredients and formulations (Beugnet and Franc, 2012). Similar advances have also been achieved in poultry medicine (EMA, 2017a; Petersen et al., 2021; Sari et al., 2022). Increasing awareness of arthropod-borne diseases and the advantages of oral administration have led to the development of a new class of compounds – the isoxazoline derivatives (Shoop et al., 2014).

**Fig. 1:**
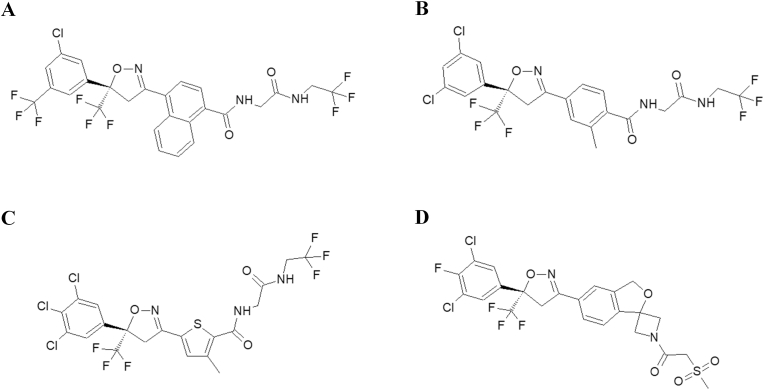
Chemical structures of afoxolaner (A), fluralaner (B), lotilaner (C) and sarolaner (D).

Currently, four isoxazolines are approved for use: afoxolaner, fluralaner, sarolaner, lotilaner, available in various products for dogs and cats (EMA, 2014a; 2014b, 2015a; 2017b). All those compounds share a similar chemical structure (Fig. 1). The most recent addition to this group is esafoxolaner, the S-enantiomer of afoxolaner. Esafoxolaner received its first marketing authorization in the European Union in 2021 as a component of a combination product (EMA, 2020).

Isoxazoline derivatives constitute a unique group of antiparasitic agents introduced into veterinary practice within the past 15 years. Afoxolaner was approved in 2013 in the United States and one year later in the European Union (FDA, 2013; EMA, 2014b). In 2014, both the FDA and EMA also approved fluralaner (EMA, 2014a; FDA, 2014). Subsequent compounds were introduced in the following years: sarolaner in 2015 in the European Union and in 2016 in the United States, and lotilaner in 2017, followed by the introduction of the S-enantiomer of afoxolaner (esafoxolaner), rather than a distinct chemical entity, as part of combination veterinary products approved by the EMA in 2020 (EMA, 2015b; 2017b, 2020; FDA, 2016). These agents are characterized by a rapid onset of action, prolonged therapeutic efficacy after a single dose, and high effectiveness (Cherni et al., 2016).

Currently, fluralaner is registered for use in dogs (oral tablets, spot-on, and subcutaneous injectable solution), in cats (spot-on), in poultry, sheep (oral, drinking water) and for cattle (pour-on) (EMA, 2014a; 2017a; MAPA Brazil, 2022; da Costa et al., 2023; Reckziegel et al., 2024; APVMA, 2025). Sarolaner is administered to dogs as oral tablets and to cats as a spot-on solution (EMA, 2015a). Lotilaner has been approved for oral administration in both dogs and cats, and since 2023, also as an ophthalmic solution (Xdemvy) for the treatment of blepharitis in humans caused by *Demodex* (EMA, 2017b; FDA, 2023). Afoxolaner is available exclusively as oral tablets for dogs, its pure isomer S-afoxolaner is marketed in combination spot-on formulations for cats (EMA, 2014b; 2020). A detailed list of all approved isoxazoline-containing products, categorized by target species, is provided in the Supplementary Table 1.

One of the most widely used products is Bravecto (fluralaner), available in over 80 countries, including the United States, European Union member states, Australia, and Latin America. Due to its long duration of action (up to 12 weeks), it has gained broad popularity. In addition to conventional chewable formulations, long-acting injectable formulations of fluralaner have also been developed, providing protection against ectoparasites for up to 12 months (e.g., Bravecto Quantum in the United States, Bravecto injectable in the European Union, and Bravecto 365 in Latin America) (EMA, 2023; MIDA, 2024; FDA, 2025). Although exact global sales data for isoxazoline compounds are not publicly available, fluralaner-containing products are widely used worldwide in ectoparasite control; notably, according to MSD Animal Health, more than 350 million doses of Bravecto have been distributed in over 100 countries since its introduction in 2014 (MSD Animal Health, 2025). However, despite this extensive use and the large number of administered doses, reports of adverse effects – previously considered rare – are being increasingly documented (Palmieri et al., 2020). This growing body of evidence highlights the need for a more comprehensive evaluation of the safety profile of isoxazoline compounds.

Given the widespread and expanding use of isoxazoline derivatives, the diversity of their chemical and stereochemical features, the marked variability in their pharmacokinetic profiles, and the growing body of evidence concerning neurological adverse events, residue deposition in food-producing animals, and environmental persistence, a consolidated evaluation of current knowledge has become essential. Accordingly, the aim of this review is to critically synthesize existing data on the pharmacology and toxicology of fluralaner, afoxolaner, sarolaner, and lotilaner, encompassing their chemical properties, mechanisms of action, pharmacokinetics, safety profiles, residue behavior, and ecotoxicological risks. By integrating these perspectives, the review seeks to provide a coherent framework for interpreting the benefits and limitations of these compounds and to clarify the key areas where further investigation is required.

## 2 Chemistry of isoxazoline compounds

Fluralaner, lotilaner, afoxolaner, and sarolaner are fluoro- and chloro-organic compounds belonging to the isoxazoline class of ectoparasiticides. These molecules are highly lipophilic, have molecular weights exceeding 500 Da, and are poorly soluble in water, but dissolve readily in solvents such as dimethyl sulfoxide (DMSO) or dimethylformamide (DMF). (Table 1).

**Table 1:** Selected physicochemical properties of isoxazoline compounds.

Property	Fluralaner	Afoxolaner	Sarolaner	Lotilaner
**Molecular formula**	C_22_H_17_Cl_2_F_6_N_3_O_3_	C_26_H_17_ClF_9_N_3_O_3_	C_23_H_18_Cl_2_F_4_N_2_O_5_S	C_20_H_14_Cl_3_F_6_N_3_O_3_S
**Molecular weight (g/mol)**	556.3	625.9	581.4	596.8
**Water solubility**	0.082 mg/L (very low)	Insoluble	Not reported (likely very low/insoluble)	Insoluble
**Solubility in organics**	Soluble in DMSO and DMF (∼30 mg/mL)	Slightly soluble in DMSO, ethanol	Slightly soluble in DMSO, ethanol	Slightly soluble in DMSO, ethanol
**Appearance**	White to off-white powder/solid	White to off-white powder/solid	White to off-white powder/solid	White to off-white powder/solid
**XlogP3-AA**	5.6	6.7	3.4	6.6

A key structural feature of these agents is the presence of fluorine atoms, which profoundly influence their chemical and pharmacological properties. Naturally occurring organofluorine compounds are rare; *Streptomyces cattleya*, for example, produces them via the enzyme fluorinase (O'Hagan et al., 2002). Beyond such isolated cases, organofluorine chemistry is largely synthetic and underpins a wide array of applications, including pharmaceuticals, agrochemicals, polymers, refrigerants, lubricants, and surfactants.

## 3 The role of fluorine in drug design

The carbon–fluorine bond is one of the strongest in organic chemistry, with the bond energy higher than C-C or C-H. The corresponding bond energies (kJ/mol) are as follows: C-C 332, C-H 415, and C-F 486. Bond dissociation energy depends also on neighboring atoms and functional groups in the molecule, however regardless of the context, the C–F bond usually has a higher dissociation energy than C-H (Lemal, 2004). This makes organofluorine compounds highly stable and resistant to oxidation. Fluorine's high electronegativity alters electron distribution when it replaces hydrogen, often modifying the reactivity of nearby functional groups. For example, acetic acid has a pKa of 4.8, whereas trifluoroacetic acid exhibits a pKa of 0.2 due to the strong electron-withdrawing effect of fluorine atoms (Garavagno et al., 2024). The van der Waals radius of fluorine (1.47 Å) is only slightly larger than that of hydrogen (1.20 Å), which means the size of substituted molecules does not significantly change. This plays a key role in drug design, as fluorine substitution does not disrupt steric properties of molecules or their affinity to receptor proteins or enzymes. Fluorinated drugs typically exhibit increased stability, longer half-life in the body, higher biological activity, and better membrane permeability compared to their hydrogen analogues. The presence of fluorine can also suppress susceptibility of certain molecules to demethylation, hydroxylation, and oxidation (e.g., ezetimibe) (Henary et al., 2024), allowing the design of drugs with extended activity and reduced active dose. Fluorination of methyl groups can improve membrane permeability by increasing lipophilicity (e.g., triazoles) (Ullah et al., 2022). These characteristics make fluorination a cornerstone strategy in the design of modern pharmaceuticals. Isoxazoline derivatives fulfill these criteria, and their fluorinated structures strongly influence their pharmacological efficacy and toxicological profile.

## 4 Chirality, biological activity, and enantiomeric selectivity of isoxazolines

Isoxazoline derivatives contain a chiral center at position 3 of the isoxazoline ring. The presence of this chiral carbon gives rise to two optical isomers, or enantiomers, which are non-superimposable mirror images of each other. These enantiomers rotate plane-polarized light in opposite directions (levorotation and dextrorotation) and are designated as *S* and *R* or *D* and *L*, depending on the nomenclature system. A 1:1 mixture of both forms is referred to as a racemate.

Although enantiomers possess identical chemical properties, their biological activities can differ dramatically. For instance, L-glucose, though sweet, is not metabolized by humans (Wright et al., 2011), and the two optical forms of sotalol differ in pharmacological action: L-sotalol functions as a β-blocker, whereas D-sotalol shows antiarrhythmic activity (Funck-Brentano C, 1993). In more extreme cases, such as thalidomide, opposite enantiomers have distinct and even adverse effects – the R-enantiomer exhibits anticancer properties, while the S-enantiomer is teratogenic and causes fetal malformations (Tokunaga et al., 2018). These examples illustrate how stereochemistry directly determines pharmacological outcomes, a principle highly relevant to isoxazoline derivatives.

In nature, most biomolecules exist in a single enantiomeric form (e.g., amino acids, sugars), and biological macromolecules such as proteins typically interact selectively with one stereoisomer. Recognition of these functional differences led the U.S. Food and Drug Administration (FDA) in 1992 to issue guidelines requiring separate evaluation of enantiomers at early stages of drug development (FDA's Policy, 1992).

The individual enantiomers of isoxazoline compounds also differ significantly in biological activity (McComic et al., 2026). S-fluralaner is 33 - 39 times more active against certain parasites than the R-form (Zhang et al., 2020), while S-sarolaner exhibits high efficacy against fleas and ticks, in contrast to the inactive R-enantiomer (McTier et al., 2016; Zheng et al., 2025). Similarly, studies conducted by Boehringer Ingelheim demonstrated that only S-afoxolaner was active against *Ctenocephalides felis*, whereas the R-form was inactive, and in the study by Prullage et al. (2021), S-afoxolaner showed high efficacy against *Ixodes ricinus* and *Ixodes scapularis* (Beugnet, 2021; Soré et al., 2025). This stereoselectivity is likely associated with the higher affinity of S-enantiomers for GABA receptors, whereas R-forms exhibit minimal receptor binding. Such stereochemical specificity directly contributes to the distinct pharmacological and toxicological profiles observed among veterinary isoxazolines (Zheng et al., 2025; McComic et al., 2026).

Consequently, the use of purified S-enantiomers may contribute to improved pharmacokinetic predictability and reduced variability, as reported for enantioselective isoxazoline compounds (Zheng et al., 2025). The selective use of active S-enantiomers is also reflected in the development of veterinary medicinal formulations. For example, Credelio tablets from Elanco contain pure S-lotilaner (Soré et al., 2025). Similarly, S-afoxolaner is used as the active enantiomer in the topical combination product NexGard Combo for cats, whereas other formulations, such as NexGard and NexGard Spectra, contain the racemic mixture of afoxolaner.

Given these pronounced enantioselective differences, separate assessment of S and R enantiomers in pharmacokinetic and toxicological studies is essential. Failure to distinguish between them may lead to misinterpretation of therapeutic concentrations or safety margins. The selective use of biologically active S-enantiomers, as seen in veterinary isoxazolines, represents a significant advance in rational antiparasitic drug design.

## 5 Chemical preparation of enantiomers

Producing single enantiomer drugs presents multiple challenges, and several chemical and physical methods have been developed to obtain pure or enriched forms. Common strategies include asymmetric synthesis, enrichment of racemic mixtures, and purification through preparative chromatography.

The development of stereoselective synthesis has twice been recognized with the Nobel Prize in Chemistry. In 2001, W.S. Knowles, R. Noyori, and K.B. Sharpless were awarded for catalytic enantioselective synthesis, and in 2021 B. List and D.W.C. MacMillan were honored for the development of asymmetric organocatalysis (Nobel Prize Committee, 2001; Nobel Prize Committee, 2021). These achievements revolutionized modern chiral drug synthesis.

In pharmaceutical manufacturing, the choice of production method must balance efficiency, yield, and cost (economic aspects are increasingly important in current drug design and production). Chromatographic separation is rarely used on an industrial scale due to high costs, whereas catalytic chiral synthesis is generally preferred for large-scale production. Isoxazoline derivatives are relatively new compounds, introduced less than two decades ago, and their manufacturing remains under patent protection, particularly concerning the enrichment of S-stereoisomers (ie. US Patent US8466115B2, WIPO Patents WO2022016490A1 and WO2017176948A1). This contributes to their relatively high prices, though cost reductions are expected following patent expiry and the introduction of generic formulations.

## 6 Mode of action of Isoxazoline compounds

The primary mechanism of action of isoxazoline derivatives involves non-competitive binding to γ-aminobutyric acid (GABA) - and L-glutamate-gated chloride channels in the nervous system of arthropods, leading to inhibition of chloride ion influx and disruption of neuronal signaling (Gassel et al., 2014; Weber and Selzer, 2016). This results in sustained neuronal excitation, hyperactivity of the nervous system, and ultimately paralysis and death of the parasite (Kuntz and Kammandiminti, 2017).

Importantly, the mechanism of action of isoxazolines differs from that of earlier classes of ectoparasiticides. These compounds display much higher selectivity for arthropod receptors compared to their mammalian counterparts, including those of humans (Weber and Selzer, 2016). Gassel et al. (2014) demonstrated that fluralaner binds to arthropod GABA receptors with significantly greater affinity than to mammalian receptors, a property that underlies its wide safety margin. This receptor selectivity is considered a key factor contributing to the insecticidal and acaricidal efficacy of isoxazolines while minimizing neurotoxic risk in mammals.

Sager et al. (2025) investigated the electrophysiological effects of several isoxazolines on GABA receptors in humans and dogs, showing that sarolaner, afoxolaner, and fluralaner inhibited receptor activity, whereas lotilaner exhibited minimal effect, suggesting a potentially higher neurological safety profile. Furthermore, Bouzat (2012) and Sieghart (2015) demonstrated that fluralaner can interact in vivo with members of the Cys-loop receptor family in vertebrates, which are primarily expressed in the central nervous system. This interaction may explain the observations of Gaens et al. (2019), who reported transient neurological symptoms – such as generalized ataxia, myoclonus, tremors, muscle contractions, and dysphagia – in dogs, coinciding with the expected time of peak plasma fluralaner concentration. The authors suggested that these effects could result from off-target receptor inhibition.

Although such adverse effects remain rare, these findings highlight the need for further research into the relationship between the mechanism of action and the safety profile of isoxazoline derivatives in veterinary practice.

## 7 Spectrum of activity of veterinary isoxazolines

Fluralaner exhibits broad ectoparasiticidal activity in dogs, cats, and poultry. In dogs, a single oral or topical administration provides up to 12 weeks of control of flea infestations caused by *Ctenocephalides felis* and other *Ctenocephalides spp*., as well as multiple tick species including *Ixodes ricinus, Dermacentor reticulatus, Dermacentor variabilis,* and *Rhipicephalus sanguineus* (European Commission Bravecto, 2014a; 2016; Gassel et al., 2014; Fourie et al., 2015; Djuric et al., 2019). Beyond its activity against fleas and ticks, fluralaner demonstrates therapeutic efficacy against several mite infestations. Treatment of generalized demodicosis caused by *Demodex canis* has been reported (Fourie et al., 2015; Djuric et al., 2019), while high efficacy has also been documented against *Sarcoptes scabiei var. canis* and the ear mite *Otodectes cynotis* (Taenzler et al., 2016, 2017). In cats, topical fluralaner provides up to 12 weeks of protection against *C. felis* and *I. ricinus,* and is effective in eliminating *O. cynotis* infections (EMA, 2016; Taenzler et al., 2017). In poultry, fluralaner administered via drinking water (Exzolt®) is effective against the poultry red mite *Dermanyssus gallinae* in commercial laying hens, with substantial reductions in mite loads and no adverse effects on egg production (EMA, 2017a; Huyghe et al., 2017; Soares et al., 2022).

Afoxolaner, approved for use in dogs, has documented activity against *C. felis* and *C. canis* for approximately five weeks following a single oral dose, and is also indicated for flea allergy dermatitis control (European Commission NexGard SPC, 2014; Beugnet et al., 2016). Its tick spectrum includes *I. ricinus, D. reticulatus,* and *R. sanguineus* (Beugnet et al., 2016). Afoxolaner further demonstrates efficacy against *Demodex canis* in generalized canine demodicosis (Beugnet et al., 2016), *S. scabiei var. canis* (Hampel et al., 2018), and *O. cynotis* (Carithers et al., 2016), highlighting its broad acaricidal activity.

Sarolaner, licensed for dogs (Simparica®), provides one month of protection against *C. felis* and *C. canis,* with rapid onset of adulticidal activity (Simparica SPC, Zoetis, 2024; McTier et al., 2016). Its tick spectrum includes *D. reticulatus, I. hexagonus, I. ricinus*, and *R. sanguineus* (Six et al., 2016; Simparica SPC, Zoetis, 2024). Sarolaner also shows efficacy against generalized demodicosis and *O. cynotis* in controlled studies (Six et al., 2016; Becskei et al., 2018), as well as *S. scabiei var. canis* (Simparica SPC, Zoetis, 2024). In cats, the combination product containing sarolaner (Stronghold Plus®) is approved for the treatment of *C. felis, I. ricinus*, and *O. cynotis* (Stronghold Plus SPC, Zoetis, 2017).

Lotilaner, approved for dogs and cats (Credelio®), provides one month of control of *C. felis* and *C. canis*, and is active against several tick species including *I. ricinus, I. hexagonus*, *D. reticulatus*, and *R. sanguineus* following oral administration with food (Credelio SPC – dogs, Elanco, 2017; Credelio SPC – cats, Elanco, 2018; Cavalleri et al., 2017; Cavalleri et al., 2018). Lotilaner is also effective against canine demodicosis caused by *Demodex spp*. after repeated monthly dosing (Snyder et al., 2017). In human medicine, a 0.25% ophthalmic lotilaner formulation (Xdemvy®) was recently approved for the treatment of *Demodex folliculorum* –associated blepharitis (FDA Xdemvy label, 2023).

Esafoxolaner, the active isomeric component of the feline combination product NexGard Combo®, exhibits activity comparable to S-afoxolaner. It is approved for the treatment and prevention of *C. felis*, as well as for the control of *I. ricinus, I. scapularis,* and *R. sanguineus* infestations in cats (NexGard Combo, 2023; Tielemans et al., 2021). The product is also indicated for the treatment of *O. cynotis* and for mild to moderate notoedric mange caused by *Notoedres cati* in cats (NexGard Combo SPC, Boehringer Ingelheim, 2023).

Together, these data demonstrate that veterinary isoxazolines share a broad but species-specific spectrum of activity against fleas, ticks, and several mite species of clinical relevance. Differences in duration of efficacy, target parasite range, and approved host species reflect variations in molecular properties, formulation types, and regulatory submissions rather than fundamental differences in mode of action. Comprehensive evaluation of these indications is essential for their appropriate application in clinical practice.

## 8 Pharmacokinetics

The distinctive pharmacokinetic properties of isoxazolines – including absorption, distribution, metabolism, and elimination – underlie their prolonged clinical efficacy. Despite structural similarities among members of this chemical class, their pharmacokinetic parameters vary considerably.

Following oral administration in dogs, the reported bioavailability values are 26% for fluralaner (Kilp et al., 2014), 85% for sarolaner (McTier et al., 2016), 73.9% for afoxolaner (Letendre et al., 2014), and 25% for lotilaner (Toutain et al., 2017). Walther et al. (2014) demonstrated that the absorption of fluralaner is enhanced and more predictable when administered with food. This observation is consistent with the general behaviour of lipophilic orally administered drugs, whose absorption may increase under fed conditions owing to improved dissolution and solubilisation within the gastrointestinal tract. A similar pattern was observed for lotilaner, whose bioavailability increased markedly in fed animals – from 24% to 82% in dogs and from 8.4% to 100% in cats (Toutain et al., 2018). These findings indicate that feeding conditions may substantially influence the systemic availability of some orally administered isoxazolines and should therefore be taken into account when interpreting interspecies pharmacokinetic differences and expected clinical efficacy. In poultry, however, the situation appears to be different. Sari et al. (2022) demonstrated that neither feed intake nor water hardness significantly influenced the pharmacokinetic profile of fluralaner in laying hens administered via drinking water, including under fed conditions. This suggests that fluralaner administration in laying hens is relatively insensitive to variations in feeding conditions and water hardness, which is of practical importance in commercial poultry production systems. Nevertheless, the pharmacokinetic behaviour of fluralaner at the level of absorption is not directly comparable between species such as dogs and poultry. This is largely attributable to fundamental differences in feeding systems and diet composition. Diets of dogs and cats are typically rich in fat and protein, whereas poultry feed differs substantially in composition, which may influence gastrointestinal processing of the compound. Proteins may exhibit sorptive properties, while lipids can enhance the solubility of lipophilic molecules such as fluralaner, thereby affecting its absorption. In addition, differences in pharmaceutical formulation may further contribute to interspecies variability. In poultry, fluralaner is administered as a liquid formulation via drinking water, whereas in dogs it is typically provided as a solid oral dosage form (chewable tablet), which may also influence its pharmacokinetic profile.

The reported maximum plasma concentrations (Cmax) also differ among compounds: 1.7 μg/mL for afoxolaner, 3.4 μg/mL for fluralaner, 1.1 μg/mL for sarolaner, and 1.5 μg/mL for lotilaner (Letendre et al., 2014; Kilp et al., 2014; McTier et al., 2016; Toutain et al., 2017). The time to reach Cmax (Tmax) varies from 2 to 6 h for afoxolaner, approximately 24 h for fluralaner, <24 h for sarolaner, and 2 – 4 h for lotilaner (Kilp et al., 2014). This variability likely reflects formulation-related challenges associated with highly lipophilic molecules exhibiting low aqueous solubility and limited solubility in organic solvents, together with the influence of enterohepatic recirculation described for fluralaner, sarolaner, and lotilaner (Kilp et al., 2014; EMA, 2017; 2017b; Toutain et al., 2017). Overall, the oral bioavailability of isoxazolines ranges from 8.4% to 100%, depending on feeding conditions, whereas topically administered formulations show approximately 25% systemic bioavailability (Zhou et al., 2022). These findings emphasize the need for precise dosing recommendations and discourage extrapolation between compounds or across species.

Isoxazolines share several pharmacokinetic characteristics, including extensive plasma protein binding (>99.9%), low clearance rates, and large volumes of distribution - approximately 3 L/kg for afoxolaner, fluralaner, and sarolaner, and around 6 L/kg for lotilaner (Kilp et al., 2014; McTier et al., 2016; Toutain et al., 2017; Zhou et al., 2022). These properties, largely attributable to the molecular lipophilicity of the compounds, favor accumulation in lipid-rich tissues. This feature explains their prolonged efficacy but may also contribute to potential accumulation within the central nervous system and the risk of neurotoxic effects (Palmieri et al., 2020).

Elimination half-life is a key pharmacokinetic parameter determining the prolonged clinical efficacy of isoxazolines. Reported values are approximately 16 days for afoxolaner (Letendre et al., 2014), 12 - 15 days for fluralaner (Kilp et al., 2014), and up to 30 days for lotilaner (Toutain et al., 2017), based on both oral and intravenous pharmacokinetic data, whereas values for sarolaner (11 - 12 days) have been determined following oral administration only (McTier et al., 2016). In dogs and cats, fluralaner exhibits a dose-dependent half-life, which contributes to its prolonged duration of efficacy (Rakotonanahary et al., 2017). Kilp et al. (2014) also suggested that enterohepatic recirculation may further prolong the persistence of fluralaner in the body by enabling reabsorption of drug excreted in bile.

After oral administration, all four isoxazolines are eliminated primarily in faeces as unchanged parent compounds, with only a minor contribution from metabolites (European Medicines Agency and Committee for Veterinary Medicinal Products, 2012, European Medicines Agency, 2014a, European Medicines Agency, 2017b; EMA, 2015a; Toutain et al., 2018). The rate of elimination may vary between species – for example, rats eliminate fluralaner more rapidly than dogs (European Medicines Agency, 2014a, European Medicines Agency, 2014b). This limited biotransformation was further supported by Gonzalez et al. (2017), who showed that more than 90% of fluralaner is excreted unchanged, and by Pittermannová et al. (2021), who suggested that the compound undergoes only minimal metabolism.

Given that the pharmacokinetics of fluralaner, afoxolaner, and lotilaner have also been investigated following intravenous administration (Kilp et al., 2014; Letendre et al., 2014; Toutain et al., 2017, 2018), it is noteworthy that only fluralaner is currently available as a long-acting subcutaneous formulation (EMA, 2023). This formulation substantially modifies its pharmacokinetic profile, particularly with respect to prolonged systemic exposure and duration of activity. Regulatory data indicate gradual absorption from the injection site, a median Tmax of 37 days, and a mean terminal half-life of 130 days, with plasma concentrations remaining within the therapeutic range for up to 12 months after administration (EMA, 2023). Moreover, unchanged fluralaner following subcutaneous administration is excreted predominantly in faeces, whereas renal elimination appears to play only a minor role, consistent with the elimination pathway observed after oral administration, as the route of administration primarily affects the rate and extent of elimination rather than its primary route (EMA, 2023). This prolonged systemic exposure is consistent with the intrinsic pharmacokinetic properties of fluralaner observed after intravenous administration, including low plasma clearance, a relatively large apparent volume of distribution, and a prolonged terminal half-life, all of which support extensive tissue distribution and slow elimination (Kilp et al., 2014, 2016).

In summary, the pharmacokinetics of isoxazolines – characterized by extensive protein binding, low clearance, and long elimination half-lives – account for their prolonged therapeutic efficacy. However, these same features may also contribute to tissue accumulation and extended persistence of adverse effects, underscoring the importance of further studies linking pharmacokinetic behavior with safety outcomes in veterinary use. Moreover, the pharmacokinetic behavior of these compounds has potential implications for public health. Fluralaner, for instance, is approved for use in laying hens, where its high lipophilicity and prolonged half-life may result in accumulation in eggs and edible tissues. Consequently, the persistence of isoxazolines in food-producing animals warrants careful monitoring, as residue deposition could impact consumer safety. In this context, pharmacokinetic data for isoxazolines in other species remain limited. This is particularly relevant in the context of off-label use of isoxazoline derivatives in species other than those for which they are currently registered. In horses, Morgan et al. (2025) demonstrated that orally administered fluralaner at doses of 10 and 25 mg/kg was safe, well tolerated, and remained detectable in plasma for approximately 42 days. In pigs, the available data are considerably more limited and primarily concern afoxolaner, for which therapeutic efficacy against mange has been reported (Smith et al., 2020). In rabbits, several isoxazoline derivatives, including lotilaner, afoxolaner, and fluralaner, have shown high efficacy against ectoparasitic infestations (Sheinberg et al., 2017; Romero Núñez et al., 2020; Machado et al., 2025).

Taken together, these findings indicate growing interest in the use of isoxazolines beyond their conventional indications in dogs, cats, and poultry. However, the availability of pharmacokinetic data in other species remains clearly limited or, in some cases, absent. Therefore, further evaluation of withdrawal periods and residue kinetics is warranted in food-producing animals (hens, cattle), including those not yet established as target species. These aspects highlight the need for continued evaluation of withdrawal times and residue kinetics in species intended for human consumption.

## 9 Drug resistance

Experimental evidence indicates that resistance to fluralaner can be selected under laboratory conditions in non-target arthropods. Norris et al. (2023) demonstrated that repeated selection of *Musca domestica* with fluralaner resulted in the development of a resistant strain, characterized by reduced susceptibility compared with unselected populations. Subsequent mechanistic investigations indicated that resistance in this model was associated with decreased cuticular penetration, consistent with reduced internal exposure to the compound (Scott et al., 2024). In parallel, Chen et al. (2025) reported cytochrome P450-mediated cross-resistance to fluralaner and isocycloseram in *Myzus persicae*, identifying CYP6CY3 as a key metabolic determinant of reduced fluralaner sensitivity.

In the currently available scientific literature and major public databases, reports explicitly addressing resistance to veterinary isoxazolines remain scarce and where documented, pertain primarily to fluralaner (Norris et al., 2023; Scott et al., 2024; Chen et al., 2025). At present, there are no published reports documenting confirmed resistance to fluralaner or other isoxazolines in target ectoparasites of companion animals under field conditions. Nevertheless, given the expanding and increasingly intensive use of isoxazolines and evidence that these compounds and their residues can enter and persist in the environment, environmental exposure to these substances is likely to increase. Their chemical stability and prolonged persistence may further contribute to sustained environmental presence. Under such conditions, and considering the experimentally demonstrated potential for resistance development, the emergence of resistance in target ectoparasites should be regarded as a plausible future risk rather than an established phenomenon, warranting continued monitoring and further investigation.

## 10 Safety profiles and reported adverse events of Isoxazoline compounds

Although isoxazoline derivatives have gained widespread use in veterinary medicine due to their high efficacy against ectoparasites, concerns have been raised regarding their safety profile, particularly in relation to neurological adverse events. Evidence from both experimental studies and post-marketing surveillance has contributed to a better understanding of their tolerability in animals.

Limited penetration of isoxazolines into the central nervous system has been attributed, at least in part, to the activity of efflux transporters at the blood - brain barrier, including P-glycoprotein (P-gp) (Dowling, 2006; Drag et al., 2022). This protective mechanism restricts drug accumulation in the brain under physiological conditions and is considered an important determinant of the neurological safety profile of these compounds. Although direct experimental evidence is not available for all members of the isoxazoline class, existing data indicate that P-gp–mediated efflux contributes to limited CNS exposure. In line with this interpretation, fluralaner has demonstrated a wide margin of safety even in dogs carrying the ABCB1/MDR1 mutation, indirectly supporting the role of efflux transport mechanisms in limiting CNS accumulation (Walther et al., 2014; Drag et al., 2022). Impairment of efflux function—due to genetic factors, pharmacological inhibition, or overdose—may nevertheless increase CNS exposure and thereby predispose susceptible animals to neurological adverse effects.

Under normal physiological conditions, P-gp prevents significant drug accumulation in the brain, thereby contributing to overall safety. However, in cases of overdose, co-administration with P-gp inhibitors, or in animals carrying the ABCB1/MDR1 gene mutation, this protective mechanism may be compromised, increasing the risk of neurotoxic effects such as tremors, ataxia, and seizures (Dowling, 2006; Swain et al., 2013). Additional risk factors include MDR1 mutations predisposing to drug hypersensitivity in certain dog breeds (Gramer et al., 2011), as well as individual variability in pharmacokinetic parameters such as gastrointestinal pH, gastric emptying time, intestinal transit, plasma protein binding, age, and sex (Sagawa et al., 2009).

Given their long elimination half-lives, high lipophilicity, and extensive plasma protein binding, isoxazolines may persist in the body for extended periods. These pharmacokinetic properties, while beneficial for maintaining sustained antiparasitic efficacy, they may also predispose to delayed or cumulative neurotoxic effects, particularly in animals with impaired metabolism or P-gp dysfunction. Therefore, pharmacokinetic behavior should be considered an integral component of the safety assessment of isoxazoline derivatives.

Several controlled studies have evaluated the safety of fluralaner in poultry and companion animals. Soares et al. (2022) demonstrated that oral administration of fluralaner via drinking water at the therapeutic dose (0.5 mg/kg body weight, twice at a 7-day interval) was well tolerated in hens, with no adverse effects on egg production or quality. Similarly, Huyghe et al. (2017) confirmed the safety of fluralaner in breeder hens, reporting no effects on egg count, mass, fertility, or hatchability. Prohaczik et al. (2017) showed that even doses three to five times higher than recommended caused no clinical abnormalities or reductions in egg production.

Long-term safety studies in dogs yielded comparable findings. Kuntz and Kammandiminti (2017) administered lotilaner orally once a month for 8 months at up to five times the maximum recommended dose and found no toxicologically relevant changes in clinical, hematological, or histopathological parameters. Additional investigations in non-traditional species also confirmed a favorable safety profile. Oral administration of afoxolaner (NexGard®) at 2 mg/kg in Burmese pythons caused no observable adverse effects (Gamez et al., 2020), while a single oral dose of 68 mg in pigs resulted only in mild pruritus (Bernigaud et al., 2018). Similarly, the long-acting injectable formulation of fluralaner administered in dogs was well tolerated, as demonstrated both in EMA assessment documentation and in the field study reported by Petersen et al. (2024) (EMA, 2023).

Despite promising experimental safety data, post-marketing pharmacovigilance has documented neurological adverse events associated with isoxazoline use. In an online survey of veterinarians and pet owners (“Project Jake”), Palmieri et al. (2020) collected 2751 responses, of which 1594 dogs had received a flea/tick parasiticide and 1325 an isoxazoline product. These survey findings were compared with publicly available adverse event (AE) reports from the U.S. Food and Drug Administration (FDA) and the European Medicines Agency (EMA). For the FDA database, 32 374 reportable AE in dogs were identified between January 2013 and September 2017; within these reports, death was recorded in 2.4% and 2.5% of AE associated with afoxolaner and fluralaner respectively, and seizures in 6.9% and 2.8%, while the corresponding proportions for sarolaner were 3.2% for death and 20.5% for seizures. EMA data for isoxazoline products over a longer time frame (2013 - 2019), as summarized and reported by Palmieri et al. (2020), showed markedly higher proportions of serious neurological adverse events, with the percentage of reports including death ranging from 4.76% to 28.6% and those including seizures from 9.0% to 55.1% across the four isoxazoline compounds. The authors emphasized that these figures represent the distribution of clinical signs within spontaneously reported adverse events, rather than incidence rates in the treated dog population, and that under-reporting and reporting bias likely influence both FDA and EMA datasets.

Additional evidence has been provided by Bates et al. (2024), who analyzed reports of neurological adverse effects associated with isoxazoline exposure in both dogs and cats. Their study highlighted that neurological signs such as tremors, ataxia, seizures, and altered mentation were the most frequently reported manifestations following exposure to isoxazolines, occurring across different compounds within the class. Importantly, the authors noted that these events were reported in animals both with and without a prior history of neurological disease, underscoring the variability in individual susceptibility. Consistent with pharmacovigilance data, Bates et al. (2024) concluded that while neurological adverse effects remain uncommon relative to the widespread use of isoxazolines, their occurrence warrants continued vigilance and systematic reporting to better define predisposing factors and risk profiles.

More post-marketing data from the FDA's CVM ADE Comprehensive Clinical Detail Report provide further insight into the safety profiles of these agents in clinical use. Analysis of adverse event reports (Table 2) indicates that afoxolaner is most frequently associated with reactions involving the gastrointestinal system, the skin and subcutaneous tissue, and the nervous system, which together constitute the predominant categories of submissions. Reports concern primarily dogs, reflecting the species in which the compound is most widely used. Overall, the profile of adverse event categories documented for afoxolaner aligns closely with that described for other members of the isoxazoline class.

**Table 2:** Summary of the reported frequency of adverse events associated with four veterinary isoxazoline derivatives based on data from the FDA Center for Veterinary Medicine Adverse Drug Event (CVM ADE) Comprehensive Clinical Detail Report Listing.

Side effect	Afoxolaner		Fluralaner		Lotilaner		Sarolaner		Mean
	CVM ADE Comprehensive Clinical Detail Report Listing; Cumulative Date Range: 04-Sep-2013 -thru- 31-Jul-2018		CVM ADE Comprehensive Clinical Detail Report Listing; Cumulative Date Range: 25-May-2014 -thru- 31-Jul-2018		CVM ADE Comprehensive Clinical Detail Report Listing; Cumulative Date Range: 18-Jan-2018 -thru- 31-Jul-2018		CVM ADE Comprehensive Clinical Detail Report Listing; Cumulative Date Range: 24-feb-2016 -thru- 31-jul-2018		
	Number of reports	Percentage of total (%)	Number of reports	Percentage of total (%)	Number of reports	Percentage of total (%)	Number of reports	Percentage of total (%)	Percentage of total (%)
**Gastrointestinal disorders**	15078	26.8	17771	32.7	166	33.81	2284	22.58	29.0
**Nervous system disorders icluding behavioral disorders**	11032	19.61	8968	16.5	48	9.78	3830	37.87	20.9
**Skin and subcutaneous tissue disorders**	12543	22.3	5016	9.2	37	7.54	553	5.47	11.1
**Lack of efficacy**	4965	8.83	7100	13.1	53	10.79	698	6.9	9.9
**General disorders and administration site conditions**	2787	4.95	3679	5.5	58	11.81	800	7.91	7.5
**Metabolic and nutrition disorders**	3189	5.67	2964	6.8	51	10.39	511	5.05	7.0
**Blood, lymphatic and immune system disorders**	1347	2.39	2250	3.4	29	5.91	340	3.36	3.8
**Urinary system and kidneys**	956	1.70	1848	2.0	23	4.68	242	2.39	2.7
**Respiratory, thoracic and mediastinal disorders**	1473	2.62	1094	4.1	9	1.83	195	1.93	2.6
**Hepatobiliary disorders**	1023	1.82	1357	2.5	2	0.41	232	2.29	1.8
**Eye, ear and labyrinth disorders**	937	1.67	904	1.7	7	1.43	176	1.74	1.6
**Musculoskeletal and connective tissue disorders**	741	1.32	609	1.1	4	0.81	122	1.21	1.1
**Cardio disorders**	251	0.45	452	0.8	2	0.41	51	0.5	0.5
**Vascular disorders**	62	0.11	92	0.2	1	0.2	56	0.55	0.3
**Reproductive system and breast disorders**	62	0.11	213	0.4	1	0.2	24	0.24	0.2

For fluralaner, gastrointestinal disorders represent the most frequently reported category, followed by neurological and behavioral disturbances, and thereafter by reactions affecting the skin and subcutaneous tissue. Reports of perceived lack of efficacy and general or administration-site reactions occur less commonly. Although the general distribution of categories resembles that of the other compounds, the relative weight of neurological and dermatological reports provides a characteristic profile for fluralaner within the class.

Sarolaner is most commonly associated with neurological and behavioral disturbances together with gastrointestinal disorders, which collectively form the dominant reporting categories. In dogs, these reactions predominate, whereas reports in other species remain comparatively infrequent. Overall, the distribution of adverse event categories mirrors that noted for afoxolaner, with neurological and gastrointestinal signs forming the core of reported clinical manifestations.

For lotilaner, gastrointestinal disturbances constitute the most frequently reported category, followed by general systemic or administration-site reactions and reports of perceived lack of efficacy. Less frequent categories include metabolic and nutritional disturbances, dermatological reactions, hematological or immunological findings, behavioral changes, and renal, urinary or neurological signs. Reactions involving other systems appear only sporadically within the dataset.

Taken together, the four compounds demonstrate both shared and compound-specific characteristics. Gastrointestinal reactions represent the most consistently reported category across the isoxazoline class as a whole, a finding that may reflect common physiological response patterns observed in post-marketing safety data and the widespread use of orally administered formulations. Neurological and behavioral disturbances are also frequently documented and correspond to the known pharmacodynamic targets of isoxazolines, which modulate arthropod ligand-gated chloride channels and may, under susceptible conditions, affect mammalian pathways. Fluralaner shows a comparatively prominent representation of dermatological reactions, whereas sarolaner exhibits a relatively greater proportion of neurological and behavioral reports. Lotilaner presents a more evenly distributed pattern among less frequent categories, with gastrointestinal and general systemic reactions forming its principal profile. Despite these differences, all four compounds share a broadly similar qualitative structure of adverse event reporting, likely reflecting their related mechanisms of action and shared physicochemical properties.

When the distributions of adverse event categories are aggregated across all four isoxazolines, gastrointestinal disorders emerge as the most frequently reported group, followed by neurological and behavioral disturbances, skin and subcutaneous tissue disorders, reports of perceived lack of efficacy, general systemic or administration-site reactions, and metabolic or nutritional disorders. This consolidated profile provides an integrated overview of the types of reactions most often associated with isoxazoline use. The synthesis presented here is our own analysis based on the available FDA CVM ADE Comprehensive Clinical Detail Report Listings. The discrepancy between the broad safety margins observed in controlled studies and the relatively high number of post-marketing reports suggests the presence of individual susceptibility factors. Potential contributors include genetic variability in drug metabolism, differences in blood–brain barrier permeability, and drug–drug interactions affecting pharmacokinetics.

Interpretation of these patterns must take into account several important limitations. The FDA CVM ADE datasets cover different and non-overlapping time windows for each compound: afoxolaner from 4 September 2013 to 31 July 2018, fluralaner from 25 May 2014 to 31 July 2018, lotilaner from 18 January 2018 to 31 July 2018, and sarolaner from 24 February 2016 to 31 July 2018. These discrepancies preclude direct comparison of numerical report counts, particularly for lotilaner, which has the shortest period of post-marketing observation. Furthermore, the CVM ADE database documents adverse event reports rather than the number of individual animals affected. A single animal may be associated with multiple entries, and the total number of animals exposed to each compound is unknown. As a consequence, the data permit characterisation of the relative distribution of adverse event categories between compounds, but they do not allow estimation of incidence, prevalence, or risk within the treated population. The dataset therefore provides insight into patterns of reported reactions but cannot be used to infer their true frequency in clinical practice.

In summary, while isoxazoline derivatives are generally regarded as safe and effective antiparasitic agents, veterinarians should remain alert to potential gastrointestinal and neurological adverse effects, particularly in predisposed individuals. Further studies are needed to clarify the mechanisms underlying these rare reactions in the context of the extensive global use of this drug class, and to better characterize the risk factors contributing to interindividual variability in safety outcomes.

## 11 Isoxazoline residues in animal products and public health implications

An additional safety concern regarding isoxazoline derivatives, particularly when used in food-producing animals, involves the potential presence of drug residues in edible tissues and animal-derived products intended for human consumption. Recently, Cui et al. (2024) investigated fluralaner residues in eggs from laying hens treated with therapeutic doses of fluralaner administered via drinking water on days 0 and 7. The highest mean concentration detected during a 23-day monitoring period was 1235 μg/kg on day 13 – approaching the European Union's maximum residue limit (MRL) of 1300 μg/kg established for eggs and poultry tissues (EMA, 2017).

According to Commission Regulation (EU) No. 2021/1757, the approved MRLs for fluralaner are 500 μg/kg in muscle, 1500 μg/kg in fat, 1000 μg/kg in liver, and 1300 μg/kg in eggs, reflecting the compound's lipophilic nature and extensive tissue distribution (EMA, 2017). Residue depletion studies show that fluralaner is eliminated slowly from poultry, with detectable concentrations persisting for up to two weeks, consistent with the 14-day withdrawal period recommended for meat and offal, while no withdrawal period is required for eggs under the approved conditions of use (European Medicines Agency EMA, Committee for Veterinary Medicinal Products CVMP, 2022; Cui et al., 2024).

The public health relevance of these findings becomes particularly evident when considered in the context of large-scale egg production and frequent dietary exposure. Poland, for example, is one of the major egg-producing countries in Europe, with annual production reaching approximately 10.0 - 10.3 billion eggs in 2020 - 2021, and it remains the largest poultry meat producer in the European Union, accounting for 20.5% of total EU poultry output in 2024 (Agroberichten Buitenland, 2023; Eurostat, 2025). In addition, available estimates indicate that egg consumption in Poland remains substantial, with approximately 139 - 162 eggs consumed per person annually in recent years (Agroberichten Buitenland, 2024). In such settings, even a low frequency of non-compliant use, off-label administration, or insufficient adherence to approved treatment conditions may translate into a substantial absolute number of eggs or poultry products entering the market with residue concentrations approaching regulatory thresholds.

From a toxicological and regulatory perspective, this issue is particularly relevant for food products such as eggs and poultry meat that may be consumed regularly and repeatedly over long periods. EMA and EFSA have emphasized the importance of harmonized human dietary exposure assessment for residues of veterinary medicines in food of animal origin, reflecting the relevance of chronic consumer exposure through repeated intake rather than isolated consumption events (EMA/EFSA, 2023).

Accordingly, the concern is not limited to occasional exceedances of maximum residue limits (MRLs), but also includes the possibility of sustained low-level dietary exposure if residue control or compliance with authorized conditions of use is inadequate. This may be especially relevant in populations with high habitual consumption of eggs or poultry products, particularly considering the high lipophilicity and strong protein binding of isoxazolines, which may favor their retention and accumulation in biological tissues.

In this context, the implications of residue persistence should also be considered in relation to toxicological thresholds, such as the no observed adverse effect level (NOAEL), which forms the basis for establishing the acceptable daily intake (ADI). Consequently, even low-level, long-term dietary exposure may be relevant if it approaches a significant fraction of the ADI, particularly in populations with high and habitual consumption of animal-derived products.

The issue may also extend beyond poultry. Fluralaner-based formulations have been evaluated in cattle in Brazil, where therapeutic efficacy against major ectoparasites has been demonstrated (da Costa et al., 2023), and a topical formulation has been registered for use in this species (Supplementary Table 1). In this context, the public health relevance of residue persistence should also be considered in relation to countries with high consumption of animal-derived foods. In Brazil, beef consumption remains high, at approximately 37.4 kg per capita per year, while domestic chicken meat consumption exceeds 46 kg per capita annually (ABIEC, 2024; USDA Foreign Agricultural Service, 2025).

Although residue depletion data for bovine tissues remain limited, the combination of persistent pharmacokinetic properties, expanding use in food-producing animals, and high dietary intake of animal products further supports the need for careful monitoring.

The properties that make fluralaner pharmacologically attractive - its high lipophilicity, long elimination half-life, and extensive distribution - also increase the potential for accumulation in animal-derived products. As such, the pharmacokinetic persistence of isoxazolines in food-producing animals has implications not only for veterinary safety but also for public health protection. Continued residue surveillance and further depletion studies are essential to ensure consumer safety and regulatory compliance.

## 12 Environmental toxicity and ecotoxicological risks of Isoxazoline compounds

Environmental exposure to isoxazoline derivatives has become an increasingly important component of their overall toxicological evaluation. Although these compounds are primarily administered to individual companion animals, their widespread global use (Supplementary Table 2) has raised concerns regarding unintended environmental release and potential effects on non-target organisms. This issue has gained additional relevance following the authorization of fluralaner for use in laying hens, where treatments are conducted at the flock level and may involve the simultaneous administration of the drug to thousands to tens of thousands of birds. In intensive poultry production systems – particularly in countries that are major global egg producers – the cumulative amount of fluralaner entering the environment via litter, manure, and associated waste streams may substantially exceed inputs resulting from companion animal use. Consequently, the original VICH GL6 guideline (EMA/VICH, 2000), which assumed negligible ecological risk due to the small quantities administered to individual animals, no longer adequately reflects current conditions, particularly in light of both the rapid global expansion of pet ownership and the large-scale application of isoxazolines in commercial poultry production (Diepens et al., 2022; Wells and Collins, 2022).

Isoxazolines may enter the environment through multiple pathways, including shedding from hair, excretion in urine and faeces, and direct contamination of water bodies when treated animals swim or bathe (Domingo-Echaburu et al., 2021). Residues have also been detected in shed hair used by wild birds for nest construction, indicating indirect environmental transfer, and secondary transfer between dogs has been documented (Diepens et al., 2022). Faecal excretion, however, represents the primary route of environmental exposure, with fluralaner concentrations in the faeces of treated dogs reaching up to 44 000 μg/kg – several orders of magnitude higher than levels observed in urine or hair (Diepens et al., 2022). Because fluralaner is excreted largely unchanged and persists in faeces for several weeks, dung dependent organisms such as dung beetles and coprophagous flies are likely to experience prolonged exposure (Du Toit et al., 2012).

In companion animals, environmental emissions are diffuse and difficult to quantify, and may be partially mitigated by waste collection and disposal practices. In contrast, in poultry production, fluralaner is currently the only isoxazoline in use and is administered at lower doses per kilogram of body weight; however, treatments are conducted at the flock level, and manure is routinely applied to land, resulting in direct and concentrated environmental exposure. Consequently, although total input per animal may be lower, environmental release in poultry systems is more predictable and locally intensified. These differences highlight the importance of considering not only the magnitude of use but also the structure and pathways of environmental emission when assessing the ecological risk of isoxazoline compounds.

Ecotoxicological studies indicate that many invertebrates are highly sensitive to the neurotoxic effects of isoxazolines. For fluralaner, 48-h LD_50_ values range from 2.9 to 65.6 ng per insect in species such as *Haematobia irritans* and *Tribolium castaneum*, while oral LC_50_ values for *Drosophila melanogaster* and *Aedes aegypti* range from 1.8 to 12 ppm (Wells and Collins, 2022). The compound also exhibits high acute toxicity toward *Daphnia magna* (LC_50_ = 0.047 mg/L; EMA, 2017). Lotilaner excreted by treated mice has been shown to cause significant mortality in mosquito larvae; however, no detectable toxicity to fish has been observed (Cooper, 2023). Ecotoxicity data for afoxolaner and sarolaner remain limited, restricting comprehensive assessment of their environmental hazard profiles.

Environmental risk assessment (ERA) data further indicate that fluralaner is highly persistent, with DT_50_ values of up to 196 days in aquatic sediments and very low water solubility (0.1 mg/L), both of which favor environmental accumulation (EMA, 2021). These physicochemical properties are consistent with its classification as highly toxic to aquatic invertebrates, and its ability to exert potent insecticidal effects at extremely low concentrations raises significant ecological concern (Wells and Collins, 2022). Despite its low water solubility, fluralaner may still pose a risk to aquatic organisms. This is supported by general environmental principles describing lipophilic organic compounds, which tend to accumulate in sediments and biological tissues, thereby enabling exposure even at very low dissolved concentrations (Karickhoff et al., 1979; Liu et al., 2019). Available evidence further suggests that isoxazolines, particularly fluralaner, exhibit considerable environmental stability which, in combination with slow elimination and prolonged excretion, may result in continuous input of residues into the environment. This is especially relevant for long-acting injectable formulations, where sustained release over extended periods may contribute to chronic environmental exposure and increase the likelihood of ecotoxicological effects.

The regulatory maximum residue limits (MRLs) established for fluralaner in edible tissues – discussed earlier in the context of food safety – also reflect its slow elimination, high lipophilicity, and persistence in biological matrices. From an ecotoxicological perspective, relatively high permissible residue levels, particularly in fatty tissues and eggs, underscore the extent of tissue retention and the potential for environmental dissemination through the disposal of poultry offal, manure, carcass waste, or egg-processing by products. These waste streams may act as point sources of contamination, creating exposure pathways for scavengers, decomposer organisms, and other non-target species. The recent detection of fluralaner residues approaching MRL levels in eggs from treated laying hens (Cui et al., 2024) further highlights its persistence in biological matrices. Detectable residues may remain for up to two weeks, despite the approved withdrawal period being zero days for eggs and 14 days for meat and offal.

Given the millions of doses administered annually (Supplementary Table 2), the high stability and persistence of these compounds in the environment, and residue levels indicating prolonged retention in animal tissues, there is a clear need for expanded research into environmental fate, sublethal effects (e.g., oxidative stress in fish), and broader ecological consequences (Wagner, 2020; Jiang and Old, 2025). Similar concerns have been raised for other widely used ectoparasiticides. In the case of compounds such as fipronil and imidacloprid, their widespread use and initial regulatory acceptance were followed by accumulating evidence demonstrating adverse effects on non-target organisms, particularly pollinators (Perkins and Goulson, 2023; EMA, 2023; Joachim et al., 2025). In this context, environmental risk assessments of veterinary ectoparasiticides indicate that emissions should, wherever possible, be minimized at the point of use (EMA, 2012). In practice, this includes strict adherence to approved conditions of use, avoidance of unnecessarily frequent treatments, and the use of combination products only when clearly justified (EMA, 2012; EMA, 2023). Although such measures have not yet been specifically standardized for isoxazolines, they remain consistent with general principles of environmental risk mitigation for veterinary medicinal products with the potential to affect aquatic ecosystems and dung fauna.

Although isoxazoline-based parasiticides are considered selectively safe for mammals at therapeutic doses, their impact on non-target species – particularly insects and aquatic invertebrates – raises significant concerns. Current ecotoxicological databases remain incomplete, and existing risk-mitigation strategies rely largely on responsible user behavior rather than regulatory controls (EMA, 2023). In this context, it is important to consider whether isoxazoline residues may pose a risk to non-target organisms, including both pollinators and dung-associated invertebrates, particularly given their increasing use and physicochemical properties that favor environmental persistence.

## 13 Summary and future perspectives

Historically, many chemotherapeutic agents used in veterinary medicine were initially developed for human medicine and subsequently adapted for animal use. However, parasiticides represent a distinct group, as several compounds were originally developed as agricultural insecticides and only later introduced into veterinary practice. Examples include organophosphates such as metrifonate, as well as more recent compounds such as imidacloprid and fipronil, which were first widely used in crop protection before being repurposed as ectoparasiticides in animals (Tingle et al., 2003; Jeschke et al., 2011; Sparks and Nauen, 2015).

In the case of isoxazolines, the development pathway was partially similar but ultimately divergent. These compounds were initially identified within agrochemical discovery programs aimed at developing novel insecticides with modes of action distinct from existing classes (Sparks, 2013; Selzer and Epe, 2021). However, unlike imidacloprid or fipronil, isoxazolines were not widely commercialized for crop protection. Instead, selected compounds from this class were subsequently developed and optimized for use in veterinary medicine as highly effective ectoparasiticides. At a later stage, interest has also emerged regarding their potential applications in human medicine, reflecting a broader pattern observed for certain antiparasitic agents. A notable example is ivermectin, which was first introduced in veterinary medicine and only later adopted in human medicine, including large-scale use in the control of onchocerciasis (Crump and Ōmura, 2011).

In 2023, the FDA approved the first ophthalmic preparation containing lotilaner for the treatment of *Demodex blepharitis*. Additional phase II clinical trials have been undertaken to evaluate a 2% lotilaner gel for rosacea associated with *Demodex folliculorum*, and in April 2024 recruitment concluded for another phase II study assessing oral lotilaner administration in humans. These developments underscore the potential translational value of isoxazolines beyond veterinary parasitology.

Pharmacokinetic and safety data generated across multiple animal species may provide a robust foundation for future human applications. Nevertheless, interspecies extrapolation must be approached with caution. Although fundamental pharmacokinetic processes are broadly conserved among mammals, the occurrence of neurological adverse effects reported in veterinary practice emphasizes the need for thorough evaluation of neurotoxicity, exposure thresholds, and susceptibility in humans. Further investigation into both the therapeutic potential and the mechanistic basis of neurotoxicity remains essential.

In veterinary and environmental health, several additional areas of potential application merit attention. Burgess et al. (2020) demonstrated that fluralaner exhibits potent activity against synanthropic filth flies, including horn flies, screwworm flies, and multiple strains of the house fly, suggesting its utility against insect populations exhibiting resistance to conventional insecticides. This observation is particularly relevant in light of the recent re-emergence of the New World screwworm (*Cochliomyia hominivorax*) in parts of South and Central America and on Caribbean islands – an event that has renewed global concern regarding insecticide resistance, control failures, and the need for novel parasiticides with distinct modes of action. Isoxazolines, due to their unique binding profile and high selectivity for arthropod ligand-gated chloride channels, may offer an alternative tool for integrated pest management in such contexts.

Despite these promising findings, significant knowledge gaps persist. Further research is required to characterize the toxicity of excreted residues following treatment of dogs and cats, particularly their effects on coprophagous insects such as dung beetles, as well as potential impacts on aquatic organisms. The expanding use of isoxazolines – including their emergence in human medicine – may create additional environmental entry points, amplifying the relevance of comprehensive ecotoxicological evaluation. Consequently, new environmental toxicity data are urgently needed to inform realistic ERA scenarios (Berny et al., 2024).

In summary, isoxazoline derivatives represent an important class of antiparasitic compounds with substantial and growing relevance across veterinary, environmental, and potentially human medicine. Their distinctive pharmacokinetic and pharmacodynamic properties, coupled with high efficacy, have made them indispensable tools in modern ectoparasite control. Yet their potent neuroactive mechanisms, prolonged persistence, and expanding environmental footprint highlight the need for continued, multidisciplinary research to address remaining gaps in pharmacology, neurotoxicity, residue kinetics, and ecological impact. Ensuring their safe and sustainable use will depend on refining safety assessments, guiding responsible application, and mitigating emerging toxicological and environmental risks through continued pharmacovigilance, residue monitoring, and environmental risk assessment.

## CRediT authorship contribution statement

**Paulina Markowska-Buńka:** Writing – review & editing, Writing – original draft, Visualization, Supervision, Resources, Project administration, Conceptualization. **Bartosz Rasiński:** Writing – review & editing, Writing – original draft, Visualization, Supervision, Software, Resources, Project administration, Investigation, Data curation. **Hubert Ziółkowski:** Writing – review & editing, Writing – original draft, Visualization, Supervision, Resources, Project administration, Methodology, Investigation, Data curation, Conceptualization.

## Financial support

This study was supported by a grant (No. 15.610.008-110) from the 10.13039/501100012706University of Warmia and Mazury in Olsztyn. Moreover, the study was funded by the Minister of Science under the Regional Initiative of Excellence Program.

## Declaration of competing interest

The authors declare that they have no known competing financial interests or personal relationships that could have appeared to influence the work reported in this paper.
