# Supplementary material for: Pharmacology and toxicology of veterinary isoxazolines: a review
Source: Int J Parasitol Drugs Drug Resist. 2026 Apr 16;31:100645. doi: 10.1016/j.ijpddr.2026.100645 (PMC13122240; doi:10.1016/j.ijpddr.2026.100645)
Supplement: Multimedia component 1 [file mmc1.docx]

**Supplementary Table 1.** Isoxazoline-based veterinary medicinal products.

| **Isoxazoline derivatives** | **Product** | **Active compound** | **Target species** | **Formulation** | **Region of registration** | **References** |
| --- | --- | --- | --- | --- | --- | --- |
| **Afoxolaner** | Frontpro, Nexgard | afoxolaner | dog | oral | EU, USA, South America | FDA, 2013; EMA, 2014b |
|  | Nexgard spectra | afoxolaner + milbemycin oxime |  |  | EU, South America, Australia, Asia | EMA, 2015a |
|  | Nexgard plus | afoxolaner + milbemycin oxime + pyrantel pamoate |  |  | USA | FDA, 2020a |
| **Fluralaner** | Bravecto, Fluralaner Intervet | fluralaner | dog | oral | EU, USA, Australia, worlwide | EMA, 2014a; FDA 2014 |
|  | Bravecto injectable, Bravecto Quantum, Bravecto 365 |  | dog | suspension for injection | EU, USA, Latin America | EMA, 2023; MIDA, 2024; FDA 2025 |
|  | Bravecto, Bravecto Plus |  | dog, cat | spot-on | EU, USA | EMA, 2016 |
|  | Exzolt |  | poultry | oral (drinking water) | EU | EMA, 2017a |
|  | Exzolt 5% |  | cattle | pour-on | Brazil | MAPA Brazil, 2022 |
| **Sarolaner** | Simparica, MiPet Easecto | sarolaner | dog | oral | EU, USA, South America | EMA, 2015b; FDA, 2016 |
|  | Simparica Trio | sarolaner + moxidectin + pyrantel pamoate |  |  | EU, USA | FDA, 2020b; EMA, 2020c |
|  | Stronghold Plus/Felisecto Plus | sarolaner + selamectin | cat | spot-on | EU, USA, South America | EMA, 2018 |
| **Lotilaner** | Credelio, AdTab, Lotimax | lotilaner | dog, cat | oral | EU, USA, South America | EMA, 2017b |
|  | Credelio Plus | lotilaner + milbemycin oxime | dog | oral | EU, USA | FDA, 2021 |
|  | Credelio Quatro | lotilaner + moxidectin + pyrantel pamoate + praziquantel | dog | oral | USA | FDA, 2024 |
|  | Xdemvy | lotilaner | human | ophthalmic solution 0.25% | USA | FDA, 2023 |
| **Esafoxolaner** | Nexgard Combo | esafoxolaner + eprinomectin + praziquantel | cat | spot-on | EU | EMA, 2020b |
