# Supplementary material for: Pharmacology and toxicology of veterinary isoxazolines: a review
Source: Int J Parasitol Drugs Drug Resist. 2026 Apr 16;31:100645. doi: 10.1016/j.ijpddr.2026.100645 (PMC13122240; doi:10.1016/j.ijpddr.2026.100645)
Supplement: Multimedia component 2 [file mmc2.docx]

**Supplemantary table 2.** Global sales of selected veterinary isoxazoline products based on publicly reported financial data.

| **Company** | **Product/Portfolio (isoxazoline)** | **Turnover (net sales/sales)** | **Reporting period** | **Source (public disclosure)** |
| --- | --- | --- | --- | --- |
| Merck & Co., Inc. (MSD Animal Health) | **Bravecto®** (fluralaner; entire Bravecto line) | **1.1 billion USD** (sales) | Full year 2024 | Merck Q4 & Full-Year 2024 Financial Results – Animal Health section (statement that Bravecto sales were 1.1 billion USD in 2024) and analysis of Merck Animal Health portfolio citing the same figure (MatrixBCG “Merck & Co. BCG Matrix Analysis”). ([Merck.com](https://www.merck.com/news/merck-announces-fourth-quarter-and-full-year-2024-financial-results/)) |
| Boehringer Ingelheim Animal Health | **NexGard® family** (afoxolaner-based parasiticide portfolio: NexGard, NexGard Spectra, NexGard Combo, etc.) | **1.2 billion EUR** (net sales; +17.2% YoY, currency-adjusted) | Full year 2023 | Company press release *“Boehringer Ingelheim reports strong growth in 2023 and accelerates late-stage pipeline”*; reprinted via news services – Animal Health segment section states that NexGard, the company’s largest Animal Health product, rose to 1.2 billion EUR in 2023. ([Estadão Blue Studio](https://bluestudio.estadao.com.br/agencia-de-comunicacao/releases/releases-geral/boehringer-ingelheim-reports-strong-growth-in-2023-and-accelerates-late-stage-pipeline/)) |
| Elanco Animal Health | **Credelio Quattro™** (lotilaner + endectocides; isoxazoline-based broad-spectrum parasiticide) | **100 million USD** (net sales; “blockbuster” threshold reached in <8 months after launch) | First ~8 months on the market in 2025 (U.S. launch period) | Elanco Q3 2025 results and associated press/SEC materials report that Credelio Quattro reached 100 million USD in net sales in fewer than eight months, becoming Elanco’s fastest pet health blockbuster. ([elanco.com](https://www.elanco.com/us/newsroom/press-releases/elanco-animal-health-reports-third-quarter-2025-results?utm_source=chatgpt.com)) |
